# A digital twin model incorporating generalized metabolic fluxes to identify and predict chronic kidney disease in type 2 diabetes mellitus

**DOI:** 10.1038/s41746-024-01108-6

**Published:** 2024-05-24

**Authors:** Naveenah Udaya Surian, Arsen Batagov, Andrew Wu, Wen Bin Lai, Yan Sun, Yong Mong Bee, Rinkoo Dalan

**Affiliations:** 1 Mesh Bio Pte. Ltd., 10 Anson Rd, #22-02, 079903 Singapore, Singapore; 2 Health Services and Outcomes Research, National Healthcare Group, 3 Fusionopolis Link, #03-08, 138543 Singapore, Singapore; 3 Department of Endocrinology, Singapore General Hospital, Outram Road, 169608 Singapore, Singapore; 4 Department of Endocrinology, Tan Tock Seng Hospital, 11 Jalan Tan Tock Seng, 308433 Singapore, Singapore; 5 Lee Kong Chian School of Medicine, Nanyang Technological University, 11 Mandalay Road, 308232 Singapore, Singapore

**Keywords:** Diabetes complications, Outcomes research

## Abstract

We have developed a digital twin-based CKD identification and prediction model that leverages generalized metabolic fluxes (GMF) for patients with Type 2 Diabetes Mellitus (T2DM). GMF digital twins utilized basic clinical and physiological biomarkers as inputs for identification and prediction of CKD. We employed four diverse multi-ethnic cohorts (*n* = 7072): a Singaporean cohort (EVAS, *n* = 289) and a North American cohort (NHANES, *n* = 1044) for baseline CKD identification, and two multi-center Singaporean cohorts (CDMD, *n* = 2119 and SDR, *n* = 3627) for 3-year CKD prediction and risk stratification. We subsequently conducted a comprehensive study utilizing a single dataset to evaluate the clinical utility of GMF for CKD prediction. The GMF-based identification model performed strongly, achieving an AUC between 0.80 and 0.82. In prediction, the GMF generated with complete parameters attained high performance with an AUC of 0.86, while with incomplete parameters, it achieved an AUC of 0.75. The GMF-based prediction model utilizing complete inputs is the standard implementation of our algorithm: HealthVector Diabetes®. We have established the GMF digital twin-based model as a robust clinical tool capable of predicting and stratifying the risk of future CKD within a 3-year time horizon. We report the correlation of GMF with basic input parameters, their ability to differentiate between future health states and medication status at baseline, and their capability to quantify CKD progression rates. This holistic methodology provides insights into patients’ health states and CKD progression rates based on GMF metabolic profile differences, enabling personalized care plans.

## Introduction

Diabetes has emerged as a prominent global health crisis in the 21st century. The lifespan of patients with diabetes is estimated to be reduced by 12 years due to vascular complications^1^. In a multi-ethnic population of Singapore, the prevalence of diabetes has doubled over the past four decades, resulting in the highest global prevalence of diabetic kidney failure^2^. Among non-fatal complications of diabetes, end-stage renal disease requiring dialysis stands out as a significant driver of healthcare costs^3^. It is therefore crucial to detect chronic kidney disease (CKD) early in its course, before reaching a non-reversible state.

A digital twin can be defined as a direct digital representation of an individual based on the individuals own comprehensive biological data^4^. Using this concept, a virtual replica of the vascular system in the individual can be represented based on a mechanistic model^5^. These digital twins can then be used to simulate progression of chronic diseases and to predict probable trajectories of disease development^6^. Moreover, they can inform clinicians how altering specific mechanistic components could change disease trajectories, thereby leading patients towards less severe disease states.

In recent years, digital twin technology has surged in popularity within the healthcare sector. A PubMed trend search for the keyword ‘digital twin’ unveiled a tenfold increase in mentions over the past five years. Different techniques, measurements and applications can be used to build a patient’s digital twin. In this paper, we utilize generalized metabolic fluxes (GMF) to construct personalized digital twins of patients and study the trajectory of microvascular complications leading to chronic CKD^6^.

The GMF digital twin analysis allows us to represent the long-term changes in the rates of metabolic processes in the body as a mathematical model of personalized metabolic rates. The components of the GMF (outputs) are obtained based on a best fit approach from the observed biochemical and physiological measurements (inputs) of an individual patient. Our GMF-based digital twin is used here to identify the CKD disease states of patients at baseline and predict the occurrence of CKD within a three-year period. We also comprehensively demonstrate the utility of GMF digital twins as a clinical application tool for CKD in individuals with Type 2 Diabetes Mellitus (T2DM).

## Results

### Dataset characteristics

**Fig. 1:**
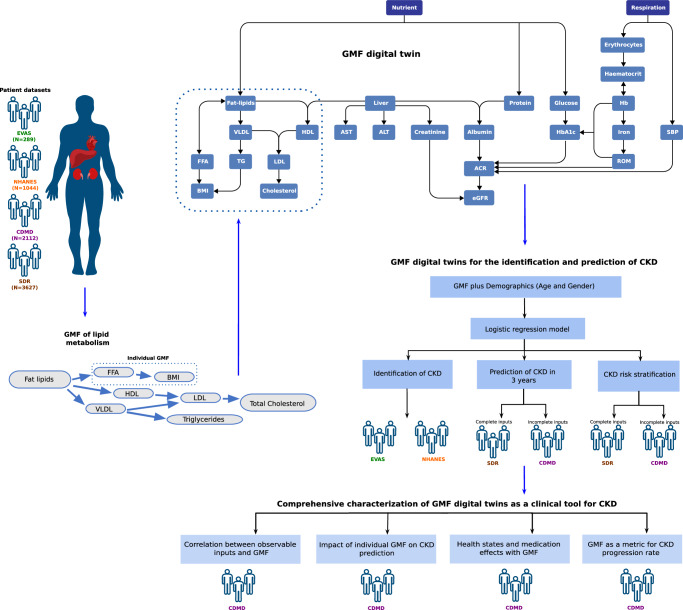
Study design. The scheme illustrates the datasets employed and how the generalized metabolic fluxes (GMF) digital twins were created and subsequently analyzed in this study. From known biological relationships, the metabolic GMF processes are constructed to form the GMF digital twins. The performance and capabilities of GMF were tested in a few analyses. We first assess its capabilities in the identification and prediction of chronic kidney disease (CKD) as well as CKD risk stratification. Subsequently, we performed a comprehensive characterization of GMF digital twins as a clinical tool for CKD.

A detailed summary of the main characteristics of each dataset is outlined in Table 1. We utilized four main datasets (EVAS, NHANES, SDR, and CDMD), two for the identification (EVAS and NHANES) and two for prediction (SDR and CDMD) of CKD. The EVAS, SDR and CDMD, datasets represented Asian-Singaporean cohorts, while the NHANES dataset represented a North America cohort. These varied datasets were chosen to showcase the versatility of GMF digital twins for analyses across different populations (Fig. 1). The EVAS dataset comprised 289 patients, of which 98 had CKD at baseline. In the NHANES dataset, there were 1044 patients, with 360 having CKD at baseline. The SDR dataset included 3627 patients, with 1420 diagnosed with CKD within 3 years, while the CDMD dataset involved 2112 patients, with 719 diagnosed with CKD in 3 years.

**Table 1:** Study population characteristics of the EVAS, NHANES, SDR, and CDMD datasets

Analysis	Identification of CKD		Prediction of CKD	
Type	Identification	Identification	Complete inputs	Incomplete inputs
Cohort	EVAS	NHANES	SDR	CDMD
Number of patients (Number of CKD positive patients at baseline/future)	289 (96)	1044 (360)	3627 (1420)	2112 (719)
Gender (M(%):F(%))	144 (49.8):145 (50.2)	534 (51.1):510 (48.9)	1917 (52.9):1710 (47.1)	1116 (52.8):996 (47.2)
Age (Mean, SD)	54 (11.1)	59 (11.9)	61.24 (11.01)	57 (12.4)
SBP, mm/Hg (Mean, SD)	133.2 (14.8)	130.3 (18.6)	132.1 (15.3)	133.8 (17.9)
BMI, kg/m2, (Mean, SD)	27.7 (5.0)	32.5 (7.5)	26.6 (5.5)	26.8 (5.6)
Hb, g/L (Mean, SD)	13.3 (1.4)	13.8 (1.6)	13.2 (1.9)	13.1 (1.7)
HbA1c, % (Mean, SD)	8.6 (1.8)	7.6 (1.9)	7.4 (1.6)	8.0 (1.8)
FBG, mmol/L, (Mean, SD)	8.9 (3.2)	8.8 (3.6)	8.1 (3.3)	8.7 (3.2)
Cholesterol, mmol/L, (Mean, SD)	4.4 (1.1)	4.6 (1.1)	4.4 (1.2)	4.5 (1.0)
HDL, mmol/L, (Mean, SD)	1.1 (0.3)	1.3 (0.4)	1.4 (0.5)	1.2 (0.4)
LDL, mmol/L, (Mean, SD)	2.5 (0.8)	2.6 (0.9)	2.5 (1.0)	2.6 (0.8)
TG, mmol/L, (Mean, SD)	1.8 (2.2)	1.6 (0.8)	1.9 (1.4)	1.5 (1.0)
Serum Creatinine, umol/L, (Mean, SD)	74.2 (26.9)	85.0 (56.3)	70.8 (23.3)	74.0 (22.7)
ACR, mg/mmol, (Mean, SD)	–	–	–	1.3 (0.8)
Serum Albumin, g/dL (Mean, SD)	–	–	4.0 (5.3)	–
ALT, U/L (Mean, SD)	–	–	33.4 (44.5)	–
AST, U/L (Mean, SD)	–	–	34.2 (45.7)	–
Hematocrit, %, (Mean, SD)	–	–	39.6 (5.2)	–

List of abbreviations used can be found in the Supplementary Materials (Supplementary Table 9).

The four datasets exhibited mostly similar characteristics, with some observed differences. Notably, the EVAS dataset included younger patients (54 [11.1]) with higher levels of glycated hemoglobin (HbA1c) (8.6 [1.8]) compared to those in the CDMD dataset (57 [12.4], 8.0 [1.8]), NHANES dataset (59 [11.9], 7.6 [1.9]), and SDR dataset (61 [11.0], 7.4 [1.6]). The NHANES dataset had slightly lower systolic blood pressure (SBP) (130.3 [18.6]), higher serum creatinine (85.0 [56.3]), and body mass index (BMI) (32.5 [7.5]) values compared to the EVAS dataset (133.2 [14.8], 27.7 [5.0], 74.2 [26.9]), the CDMD dataset (133.8 [17.9], 26.8 [5.6], 74.0 [22.7]) and the SDR dataset (132.1 [15.1], 26.6 [5.5], 70.8 [23.3]).

### GMF digital twins for the identification and prediction of CKD

**Fig. 2:**
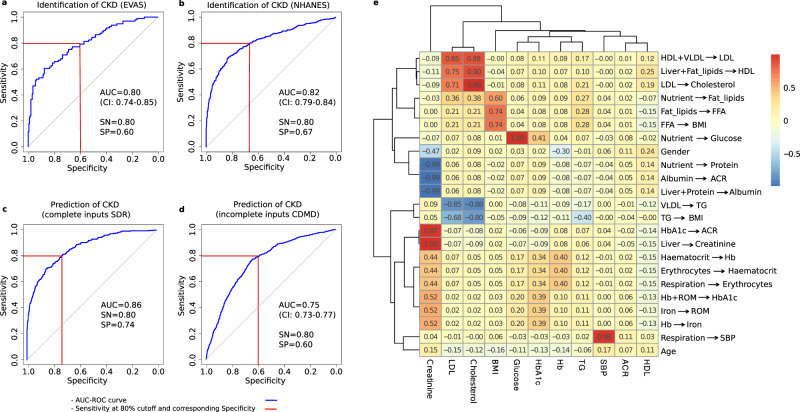
AUC-ROC curves for identification and prediction of CKD and the correlation between input parameters and GMF. **a** The identification of CKD in EVAS at baseline yielded an AUC of 0.80. **b** The identification of CKD in NHANES at baseline yielded an AUC of 0.82. **c** The prediction of CKD within 3 years in SDR with complete inputs yielded an AUC of 0.86. **d** The prediction of CKD in CDMD with incomplete inputs yielded an AUC of 0.75. The LR models for the AUC-ROC curve generations utilized the GMF values, age and gender as input parameters. The CI represents the 95% confidence interval of the AUC-ROC curve in the EVAS, NHANES and CDMD datasets. The SDR AUC-ROC curve is depicted for the Testing-2 subset of the SDR dataset, generated by the LR model trained on the SDR training set. **e** The correlation between biochemical and physiological input parameters with GMF in the CDMD dataset. The numbers in each box represent Kendall’s τ correlation value between two variables, the input parameters vs the GMF values. Stronger positive correlation is indicated by values closer to +1 and depicted in a deeper red hue. Conversely, stronger negative correlation is denoted by values closer to -1 and depicted in a deeper blue hue. These relationships are illustrated in the legend located on the right. Serum creatinine is positively correlated with a number of individual GMF, particularly the GMF related to the respiration-circulation pathways, reactive oxygen species and HbA1c production pathways. It is also negatively correlated to the individual GMF related to the albumin-ACR pathways. LDL, Cholesterol, BMI, glucose and HbA1c cluster together, whereby LDL, Cholesterol and BMI are strongly correlated to the individual GMF related to the lipid metabolism pathways. CKD chronic kidney disease, GMF generalized metabolic fluxes, AUC-ROC area under the curve receiver operating characteristic, LR logistic regression, CI confidence interval, SN Sensitivity, SP Specificity, HbA1c glycated hemoglobin, ACR albumin-creatinine ratio, LDL low density lipoprotein, BMI body mass index.

In the NHANES and the EVAS cohorts, we used baseline observations of biochemical and physiological measurements of patients (Table 2) to build individual GMF digital twins using the method described earlier^6^. Each digital twin represented the state of metabolic dynamic variables, GMF, which quantify the rates of biochemical pathways and physiological processes. Since the inputs did not include the diagnostic indicators of CKD, the estimated glomerular filtration rate (eGFR) and the urinary albumin to creatinine ratio (ACR), we first sought to test if the digital twin-based models could reflect the CKD condition, based on the evaluation of the holistic metabolic state of the patient (Fig. 1). In other terms, this analysis involved identifying the CKD state based on a set of estimated metabolic rates. For the identification of CKD, our GMF-based model achieved an AUC of 0.80 (confidence interval (CI): 0.74–0.85) in the EVAS dataset and an AUC of 0.82 (CI: 0.79–0.84) in the NHANES dataset (Fig. 2a, b).

**Table 2:** List of parameters used for GMF digital twin generation in the identification (EVAS and NHANES), complete input prediction (SDR) and incomplete input prediction (CDMD) LR models

Parameters[Units]	EVAS	NHANES	SDR	CDMD
Fasting Blood Glucose (FBG) [mmol/L]	*✓*	*✓*	*✓*	*✓*
Total Cholesterol [mmol/L]	*✓*	*✓*	*✓*	*✓*
High density lipoprotein (HDL) [mmol/L]	*✓*	*✓*	*✓*	*✓*
Low density lipoprotein (LDL) [mmol/L]	*✓*	*✓*	*✓*	*✓*
Triglyceride [mmol/L]	*✓*	*✓*	*✓*	*✓*
Serum Creatinine [*μ*mol/L]	*✓*	*✓*	*✓*	*✓*
Body Mass Index (BMI) [kg/m2]	*✓*	*✓*	*✓*	*✓*
HbA1c [%]	*✓*	*✓*	*✓*	*✓*
Hemoglobin (Hb) [g/dL]	*✓*	*✓*	*✓*	*✓*
Systolic Blood Pressure (SBP) [mm/Hg]	*✓*	*✓*	*✓*	*✓*
Albumin to Creatinine Ratio (ACR) [mg/mmol]	**X**	**X**	**X**	*✓*
Serum Albumin [g/dL]	**X**	**X**	*✓*	**X**
Alanine Aminotransferease (ALT) [U/L]	**X**	**X**	*✓*	**X**
Aspartate Aminotransferase (AST) [U/L]	**X**	**X**	*✓*	**X**
Hematocrit [%]	**X**	**X**	*✓*	**X**

Having showed that GMF digital twins could reflect and distinguish between non-CKD and CKD states among patients at the baseline time point, we examined whether the estimated metabolic rates from the GMF digital twins could predict the progression from the non-CKD to the CKD state in the future. We evaluated two logistic regression (LR) prediction models: the complete input and the incomplete input models. In the SDR dataset, where patient input values were complete, the LR model was trained on the training set and tested on two equally divided testing sets, Testing-1 and Testing-2, for evaluation. Performance metrics, including AUC, Sensitivity (SN), Specificity (SP), Negative Predictive Value (NPV), and Positive Predictive Value (PPV) for the SDR dataset, are presented in Table 3. Notably, we achieved identical AUCs of 0.86 and nearly identical values for SP, NPV, and PPV in both the testing sets, Testing-1 and Testing-2 (Fig. 2c, Table 3). We subsequently evaluated the performance of GMF digital twins in the CDMD dataset, which had incomplete patient input values (Fig. 2d). The LR prediction model here achieved an AUC of 0.75 (CI: 0.73–0.77). This underscores the robustness of GMF digital twins for the prediction of CKD.

**Table 3:** Performance metrics of the GMF-based complete input LR prediction model in SDR

Metrics	Testing-1	Testing-2
AUC	0.86	0.86
SN	80%	80%
SP	72%	74%
NPV	84%	85%
PPV	65%	66%

Model trained on the SDR training set and tested on two SDR testing sets.

Next, we explored how our predictive model stratifies patients into three different risk groups: high, moderate, and low. The distribution of the SDR and CDMD patients across the risk groups is presented in Table 4. Most patients who developed CKD in 3 years came from the high-risk group. In the SDR dataset, 62.9% of patients developed CKD in the high-risk group, whereas this fraction was 19.3% in the moderate-risk group and 5.4% in the low-risk group. The distribution of CKD cases in the CDMD dataset was overall similar: 53.3%, 17.3%, and 9.8% in the high-, moderate- and low-risk groups, respectively (Table 4). The observed frequencies in both datasets align with the expected risk frequencies, as well as the values reported in previous studies^7,8^. We also assessed the distribution of future CKD patients across CKD stages and their corresponding risk categories determined by the GMF-based prediction model in the CDMD dataset. Microalbuminuria (3.3 mg/mmol < ACR < 30 mg/mmol) was the prevailing condition among future CKD cases, constituting 93%, of which 73% were categorized as high risk (Supplementary Table 1).

**Table 4:** Risk group classification using GMF with the complete input LR prediction model (SDR Testing-2 subset) and the incomplete input LR prediction model (CDMD)

	SDR			CDMD			Risk interval
Risk group	no CKD (N)	CKD (N)	% CKD	no CKD (N)	CKD (N)	% CKD	Expected median CKD %
High (>30%)	174	295	62.9	461	526	53.3	65% (>30%)
Moderate (10-30%)	205	49	19.3	886	188	17.3	20% (10–30%)
Low (<10%)	174	10	5.4	46	5	9.8	5% (<10%)

### Correlation between the observable biochemical and physiological inputs and GMF

Utilizing the CDMD dataset, we investigated the clinical applications of GMF digital twins, extending their utility for risk prediction. We first looked at the correlation between the input parameters (biochemical and physiological) used to obtain GMF with the GMF values itself. The input parameters show strong correlation with the relevant individual GMF (Fig. 2e). Serum creatinine, which is an indicator for CKD, has a strong positive correlation with the individual GMF related to the circulation-respiratory pathways, the reactive oxygen species and HbA1c production pathways and negative correlation to the individual GMF related to the albumin-ACR pathways. Furthermore, the parameters LDL, Cholesterol and BMI show strong positive correlation to the individual GMF related to the lipid metabolism pathways.

### The influence of individual GMF impact on CKD prediction

**Fig. 3:**
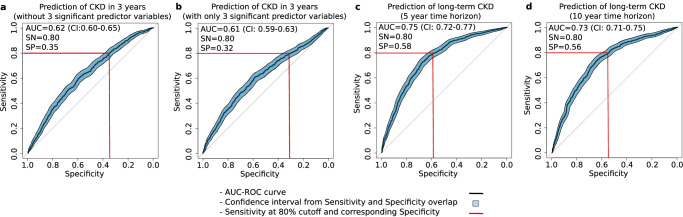
AUC-ROC curves for prediction of CKD with and without the LR significant predictor variables and for long-term CKD. **a** The prediction of CKD without the top 3 significant predictors yielded an AUC of 0.62. **b** The prediction of CKD with only the top three significant predictors yielded an AUC of 0.61. **c** The prediction of CKD in 5 years yielded an AUC of 0.75. **d** The prediction of CKD in 10 years yielded an AUC of 0.73. The LR models for the AUC-ROC curve generations utilized the non-significant predictor variables (a) or the significant predictor variables (b) or the GMF values, age and gender (c and d) as input parameters depending on the analysis in the CDMD dataset. The figure displays the CI values for Sn and Sp as filled polygons, overlapping the AUC-ROC curve along the vertical (Sn) and horizontal (Sp) axes.

After observing the correlations between the input parameters and GMF, we studied the individual GMF that were significantly impacting the CKD predictive performance based on the coefficient weights from the LR model in CDMD. Out of the 14 individual GMF and two demographic variables (age and gender) used as predictor variables for CKD prediction, the LR model revealed that two individual GMF along with gender emerged as the most statistically significant predictor variables (Table 5, *p* < 0.05). The two individual GMF were associated with HbA1c production^9^ and kidney function^10^ : Hb+ROM → HbA1c and HbA1c → ACR. Removing these three predictor variables led to a drop in prediction accuracy, yielding an AUC of 0.62 (CI: 0.60-0.65, Fig. 3a). Interestingly, using only these three predictor variables in the LR model also resulted in much lower prediction accuracy for CKD, with an AUC of 0.61 (CI: 0.59-0.63, Fig. 3b). This highlights that reducing the GMF digital twin to only a subset of statistically significant individual GMF strongly reduces predictive performance and, consequently, clinical utility.

**Table 5:** Significant predictor variables from the LR prediction model in CDMD

Predictor	Coefficient estimate	p-values
Ex.HbA1c → ACR	22.1	9.0 e-30**
Hb.ROM → HbA1c	0.2	0.036*
Gender	0.1	0.015*

Significance determined based on the LR model coefficient weight estimates and their corresponding p-values.

### Patient health states and medication effects with GMF

**Fig. 4:**
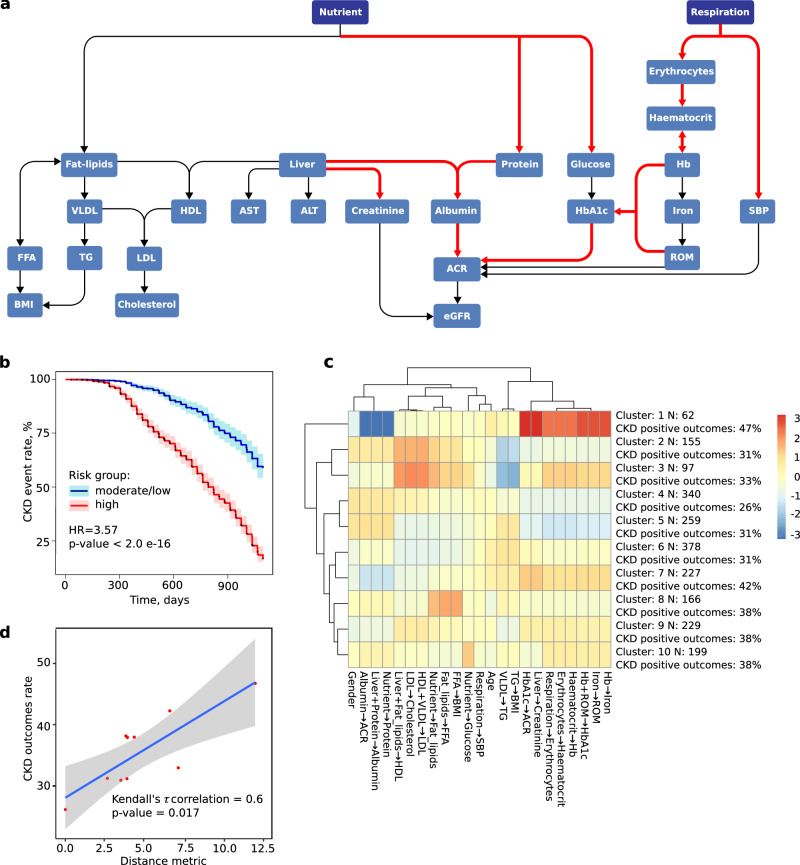
Additional analysis involving metabolic profile characterization, time-to-event analysis and patient clustering. **a** The GMF profile from a subgroup analysis of future CKD patients vs future non-CKD patients in the CDMD dataset. The GMF profile of patients who develop CKD in the future shows a poorer health profile than the GMF profile of patients who do not develop CKD in the future. This subgroup had elevated individual GMF associated with circulation, blood pressure, glucose metabolism, and kidney function. **b** The time to CKD event analysis in CDMD. Risk group 0 represents patients classified as low/moderate-risk patients whereas risk group 1 represents patients classified as high-risk patients by the GMF digital twin based logistic regression model. There is a significant difference in the event rate between the high-risk vs moderate/low- risk as evidenced by the elevated hazard ratio (HR = 3.57, *p* < 2.0 e-16). **c** Clustering pattern of patients in the CDMD dataset using GMF together with demographics. N represents the number of patients within the specific cluster, while CKD positive outcomes are calculated as the ratio of patients developing future CKD (within 3 years) to the total number of patients in that cluster. The cluster with the highest CKD outcomes rate has a distinct pattern with elevated individual GMF values related to the respiration-circulation pathways, reactive oxygen species and HbA1c production pathways and reduced individual GMF values related to the albumin-ACR pathways. The colors in each cell of the heatmap correspond to normalized values for each individual GMF or demographic within each cluster, where elevated values compared to the median are depicted in a deeper red hue, while reduced values compared to the median are shown in a deeper blue hue. This color scale is represented on the right side of the heatmap. **d** The relationship between the cluster distances and CKD outcomes rate in CDMD. There is a significant correlation (τ = 0.6, *p* = 0.017) between cluster distance as measured by the distance metric and CKD outcomes rate where the cluster with the highest CKD outcomes rate is furthest away from the cluster with the lowest CKD outcomes rate. Standard error (SE) is denoted by the gray shaded area.

To identify predictive factors for future categorization into CKD-positive or negative groups, we analyzed baseline GMF profiles. In the CDMD dataset, a subgroup analysis comparing the GMF metabolic profiles of future CKD and future non-CKD patients revealed that the future CKD group exhibited elevated individual GMF associated with circulation, blood pressure, glucose metabolism, and kidney function (Fig. 4a, Supplementary Table 2). This clearly shows that future CKD patients display a more deteriorated health state (GMF metabolic profile) compared to future non-CKD patients.

Medications, such as sodium-glucose transport protein 2 inhibitors (SGLT2i), can influence metabolic pathways, and SGLT2i is known for its role in reducing CKD risk. In this study, we investigated the impact of SGLT2i on GMF in two patient subgroups: the future CKD group and the future non-CKD group based on their baseline GMF profiles. Significant differences in individual GMF related to lipid metabolism (Fat.lipids ↔ FFA and FFA → BMI) were observed in the future non-CKD group (*p* < 0.05, Table 6). Although the effects in the future CKD group were not statistically significant but near significance (*p* < 0.1, Table 6), there was a noticeable trend involving the same lipid metabolism GMF. Our GMF-based analysis highlights the impact of medication on metabolic profiles, specifically the influence of SGLT2i on lipid metabolism, aligning with findings from prior studies^11^.

**Table 6:** Individual GMF differences in patients taking SGLT2i vs patients not on SGLT2i in the future non-CKD group and the future CKD group

Fluxes	Future Non-CKD (*p*-values)	Future CKD (*p*-values)
Fat-lipids ↔ FFA	0.008**	0.056
FFA → BMI	0.008**	0.056

Significance is assessed using p-values obtained from the Wilcoxon–Mann–Whitney *U* test.

### GMF as a metric for CKD progression rate

Having observed that GMF profiles provide insights into CKD health states as well as medication effects, our next objective was to quantify CKD progression rates in different risk groups within the CDMD dataset. To assess the rate of CKD progression in high-risk versus moderate/low-risk groups, we conducted a Kaplan–Meier time-to-event analysis and calculated the hazard ratios from the Cox-proportional hazard model. The highly significant hazard ratio of 3.6 (*p* < 0.01) indicates a markedly faster progression rate in the high-risk group compared to the moderate/low-risk group (Fig. 4b). After confirming the heightened progression rate in the high-risk patient group, we assessed the capability of GMF based digital twins to predict CKD beyond 3 years. The initial count of CKD-positive patients within 3 years was 719. Extending the prediction timeframe to 5 and 10 years resulted in a 10% and 24% increase, totaling 793 patients in 5 years and 889 patients in 10 years. The LR model’s predictive performance with GMF digital twins for long-term CKD within a 5-year and 10-year horizon yielded AUC values of 0.75 (CI: 0.72-0.77) and 0.73 (CI: 0.71-0.75), respectively. (Fig. 3c, d). This suggests that GMF digital twins is proficient in predicting CKD over extended periods, achieving AUCs similar to those obtained originally within 3 years.

Among CKD-negative patients at baseline, some profiles resembled CKD patients while others did not. As we have clearly established that GMF can be used to measure CKD progression rates as well as predict longer term CKD, we sought a metric to distinguish the profiles of patients clustered using GMF. We conducted patient clustering using two sets of variables: the first set consisted of GMF values, while the second set utilized observable inputs (biochemical and physiological). When clustering patients based on GMF values, we observed that the cluster with the highest fraction of future CKD outcomes was markedly distinct from all other clusters (Fig. 4c, a). In this cluster, the individual GMF related to circulation-respiratory pathways and the reactive oxygen, creatinine and HbA1c production pathways were increased whereas the individual GMF related to the albumin-ACR pathways were decreased. To analyze the relationship between GMF profiles and CKD development, we ordered the clusters by increasing CKD outcomes rate. We picked the cluster with the lowest fraction of positive CKD outcomes as the starting point. For all other clusters, we computed the Euclidean distance from their centers to the starting point. We examined the dependence of the CKD outcomes rate in each cluster on this distance metric. There is a significant positive correlation (*τ* = 0.6, *p* < 0.05) between the distance metric and CKD outcomes rate when we used GMF to cluster patients (Fig. 4d, b). The cluster with the highest fraction of positive CKD outcomes was the furthest away from the cluster with the lowest fraction of positive CKD outcomes. By contrast, we found no distinct cluster profiles and no significant associations between the distance metric and CKD outcomes rate when we used input parameters for clustering (Supplementary Fig. 1).

## Discussion

Current clinical guidelines recommend regular monitoring for diabetes-related complications, including cardio-metabolic and renal screenings every 4–6 months (e.g., lipid panel, HbA1c, urine ACR, eGFR), along with annual retinal exams and foot assessments for peripheral vascular disease^12^. However, these recommendations are not always followed, resulting in selective sparsity in patient data and delayed detection of complications. Current clinical indicators for kidney function, such as ACR and eGFR, are used to diagnose and stage patients with CKD^12^. Without these indicators, detecting CKD becomes challenging. Moreover, these indicators are unreliable for prognostication; ACR accurately predicts future progression in only 30% of cases, and eGFR exhibits high variability due to age and gender effects, which is also compounded by the absence of a standardized equation^13,14^. These observations underscore the current unmet clinical need for a reliable CKD identification and prediction tool in T2DM patients^15^.

To address these needs, we applied the GMF digital twin method^6^ to (i) obtain the best-fit approximation of patient health states using complete and incomplete inputs, (ii) identify CKD development in patients without relying on ACR and eGFR values, (iii) predict CKD progression, and (iv) elucidate the metabolic traits associated with high CKD progression risk as predictor variables. GMF digital twins is made up of multiple dynamic variables (from the observable inputs) characterizing the present metabolic state of a patient and mapping their future health trajectory. The principle of GMF digital twins is derived from biological information and pathways (Supplementary Table 3). To evaluate the performance of the GMF digital twins in identification of CKD at baseline (characterizing the present health state) or predicting CKD progression in the future (mapping the future health trajectory), we utilized a logistic regression (LR) model and generated corresponding AUC-ROC curves.

In retrospective clinical datasets, GMF digital twins could identify and characterize the metabolic states that corresponded to CKD (AUC = 0.80–0.82, Fig. 2a, b). Various machine learning models have been devised for CKD detection, ranging from simple methods utilizing common screening parameters to sophisticated deep learning techniques incorporating retinal imaging^16,17^. However, these approaches often suffer from either reduced identification accuracy or high complexity, necessitating additional expertise, time, and cost. GMF digital twins could accurately predict CKD progression within a 3-year period and stratify patients based on their CKD progression risk levels. When utilizing the complete set of 14 input parameters, they demonstrated high performance in predicting CKD achieving an AUC of 0.86 (Fig. 2c). Overall, the predictive performance of our model was higher and more robust than most published models that use single-time point readings (biochemical or otherwise) as their inputs^8,18–22^ (Supplementary Table 4). Moreover, our GMF digital twins successfully stratified patients based on their CKD risk scores, with the highest proportion of patients developing CKD found in the high-risk group (53.9–62.9%), consistent with findings from previous studies^7,8^. We also confirmed a significant association between ethnic diversity and future CKD prevalence and risk stratification (Supplementary Table 5 and Supplementary Table 6), which was consistent with prior observations^23^. Our GMF-based digital twin prediction model was evaluated on multiple time scales in diverse cross-country multi-center and multi-ethnic cohorts suggesting its wide applicability. These findings lay the groundwork for future clinical applications of GMF digital twins in assessing CKD risk and planning personalized care for diverse populations with T2DM.

To address the common limitation of data sparsity in clinical databases, we developed the incomplete parameter prediction model using the CDMD dataset where we utilized 11 common input parameters with missing values to construct the GMF digital twins. Despite the presence of missing parameters, this GMF-based prediction model exhibited reasonable performance with an AUC of 0.75 (Fig. 2d). We further assessed the predictive performance of GMF digital twins for CKD over extended durations within the same dataset, achieving consistent AUCs of 0.75 at 5 years and 0.73 at 10 years (Fig. 3c, d). This underscores the capability of GMF digital twins to effectively handle missing input parameters by employing a best-fit solution across multiple parameters, which remains applicable over extended time periods.

While reporting the GMF digital twins predictive capabilities, it remains crucial to identify the most significant individual GMF as predictor variables and assess their impact on the predictive performance. By analyzing the LR model, we identified three key predictor variables (two individual GMF and gender) (Table 6). Removal of these predictors from the LR model resulted in a significant drop in AUC (AUC = 0.62, Fig. 3a). Notably, the two significant individual GMF (Hb+ROM → HbA1c and HbA1c → ACR) are involved in glucose/HbA1c metabolism and kidney function, providing explainable insights into affected pathways in future CKD states. While these pathways are known to deteriorate in T2DM patients with microvascular complications, utilizing them as sole predictor variables is insufficient, as evidenced again by a substantial drop in AUC (AUC=0.61, Fig. 3b). The unique strength of GMF lies in its holistic nature, where each individual GMF is interconnected, mirroring the functions of the biological metabolic network.

Further exploration reveals additional insights from GMF. GMF digital twins inform the long-term rates of metabolic changes and predict the evolution of metabolic characteristics of microvascular complications of diabetes^6^. It can identify early indicators of disease progression to predict future health states. Our analysis reveals that patients who will develop CKD in the future exhibit an overall deteriorated metabolic profile at baseline, as indicated by elevated individual GMF or metabolic rates related to glucose production and kidney function compared to patients who do not go on to develop CKD (Fig. 4a, Supplementary Table 2). We also observed distinct GMF profile differences between patients taking SGLT2i and patients not taking SGLT2i. There were significant differences in individual lipid metabolism GMF within the future non-CKD group (*p* < 0.05, Table 6). The difference was more evident in the future non-CKD group compared to the future CKD group. We attribute this to the relatively smaller sample size of patients taking SGLT2i in the future CKD group compared to the future non-CKD group (*n* = 16 vs *n* = 23), causing the effects within the future CKD group to be non-significant but approaching significance (*p* < 0.1). This finding was consistent with earlier studies, where SGLT2i was recognized for its influence on lipid metabolism both directly^11^ and indirectly^24^. Further studies involving cohorts with a more extensive representation of patients taking SGLT2i could provide additional clarity.

GMF digital twins not only elucidate future health states but also quantify the rates of health state progression, reflecting the evolution of health. Using the time-to-event analysis, we found highly significant differences in CKD progression rates between high- and moderate/low-risk patients (HR = 3.57, *p* < 0.01, Fig. 4b). These metabolic changes in the GMF profiles may be associated with microvascular deterioration driving the increased rate of future CKD outcomes. At the initial baseline state, GMF profiles are able to cluster patients according to their metabolic characteristics which in turn reflect the degree of health deterioration. The individual GMF related to respiratory circulation, glucose metabolism and kidney function were distinctly elevated in the cluster with the highest CKD outcomes rate (Fig. 4c). Measuring the distance between cluster centers reveals a positive correlation between cluster distance and CKD outcomes rate (*p* < 0.05, Fig. 4d). The cluster with the highest positive CKD outcomes is farthest from the cluster with the lowest positive CKD outcomes. This highlights the ability of GMF to map baseline health states to patient health trajectories. In comparison, basic clinical and physiological input parameters failed to demonstrate distinct patient cluster profiles and exhibited no correlation with cluster distances and CKD outcomes rate (Supplementary Fig. 1). Overall, we could demonstrate that the explainability of our predictive model follows from the structure of GMF digital twins as personalized representations of the metabolic network. It opens opportunities for clinicians to obtain insights into key metabolic pathways leading to future dysfunctions in a given patient. This property sets our method apart from black-box machine learning approaches.

Our study has demonstrated promising results; however, it is important to acknowledge the limitations that exist. In the CDMD and SDR real-world data sets, single time-point readings for all 11 or 14 input parameters used to generate the GMF digital twins were unavailable. Consequently, we applied data aggregation to derive input parameter values which involved taking the median values of each parameter with repeated measurements over a single year. We agree that some parameters, like ACR, have high intra-individual variability^25^. In our complete model (SDR), the inclusion of ACR led to a drop in the predictive model’s performance (Supplementary Fig. 2). Due to the lack of single-day measurements for each of the parameters, stringent control over input parameters was compromised, introducing non-uniformity. Therefore, ACR was excluded from our complete prediction model (SDR) (Table 2). In our incomplete model (CDMD), we opted to include ACR. Due to the presence of missing data in the CDMD dataset, excluding an additional parameter would further reduce the model’s accuracy. Consequently, we retained ACR as an input in this model, achieving reasonable predictive performance. Secondly, our comprehensive analysis demonstrated robust results regarding the utility of GMF digital twins as a clinical application tool for CKD prediction. However, this analysis was conducted solely on the CDMD dataset, which had incomplete parameters. Additionally, certain subgroup analyses, particularly those examining medication effects, were constrained by limited sample sizes. Specialized studies on larger cohorts with complete parameters are warranted to address these limitations. Thirdly, the impact of other data forms such as omics (metabolomics, genomics, proteomics, transcriptomics), imaging, and social determinants of health on the generation of our GMF digital twins was not considered. Incorporating these aspects would enrich our GMF digital twins, enhancing their predictive accuracy and providing deeper insights into CKD pathology. Fourthly, while we compiled a comparison table of our complete inputs GMF-based model against other published models for CKD prediction (Supplementary Table 5), we did not conduct a comparative analysis with our dataset for benchmarking purposes. This was because validated open algorithms with matching endpoints and input structures were not available. As more models with similar outcomes become accessible, future studies could facilitate direct comparisons. These limitations underscore areas for future research and refinement of our approach. We seek to explore the enhanced capabilities of GMF as a metabolic digital twin application in our upcoming studies. Our future investigations aim to explore the longitudinal effects of GMF derived from multiple time points and their implications for enhancing CKD prediction accuracy. Additionally, to evaluate our GMF digital twins predictive performance in real-world settings, we plan to assess the impact of GMF-based prediction and risk stratification on patient outcomes in prospective clinical studies. These analyses were beyond the scope and objectives of the current study. Therefore, we plan to explore them in future research with an expanded study design and additional objectives.

In conclusion, we have developed a GMF-based digital twin application that successfully identifies existing metabolic pathways dysfunctional in CKD patients, and predicts future CKD progression within a 3-year time horizon. The standard implementation of our algorithm (HealthVector Diabetes®), utilizes the GMF digital twin with complete inputs. This positions our algorithm as a promising candidate for adoption in clinical settings as an identification and predictive tool for preventing CKD among T2DM patients.

## Methods

### Study design and dataset characteristics

Our study included four populations: three multi-ethnic T2DM cohorts from Singapore and one multi-ethnic T2DM North American cohort. Our first dataset (EVAS) was a multi-ethnic cohort of T2DM patients from Singapore’s Tan Tock Seng Hospital (TTSH) that were part of a clinical study and were followed-up for 5 years between 2015 to 2020^26^. The second dataset (NHANES) was obtained by selecting T2DM patients from data collected in the National Health and Nutrition Examination Survey (NHANES) that were recruited between 1999 to 2018 as part of the major National Center for Health Statistics (NCHS), Centers for Disease Control and Prevention (CDC) program^27^. The third dataset (SDR) was a group of T2DM patients extracted from a health database registry of Singapore Health Services (SingHealth), the SingHealth Diabetes Registry (SDR) between 2013 and 2020^28^. The fourth dataset (CDMD), the National Healthcare Group Chronic Disease Management Datamart (NHG-CDMD) was obtained from the electronic medical records (EMR) of TTSH, Singapore between 2008 to 2021. All patients used in the four datasets were aged between 20 and 80 years and their baseline characteristics are outlined below (Table 1). Baseline GMF metabolic profiles were calculated for each individual from the selected list of clinical and physiological parameters relevant to T2DM and CKD (Table 2). We conducted the following analyses (Fig. 1): i) identification of CKD at baseline ii) prediction of future CKD within three years iii) CKD risk stratification, iv) correlation between observable input parameters and GMF, v) impact of individual GMF on CKD prediction, vi) health states and medication effects with GMF, vii) GMF as a metric for CKD progression rate. For the identification of CKD, we used the EVAS and NHANES datasets and for the prediction of CKD and risk stratification, we used the SDR and CDMD datasets. Subsequently, for all the other analyses, we used the CDMD dataset. Ethics approval was obtained from the Singaporean (NHG and SingHealth) Institutional Review Board (IRB), EVAS (DSRB Ref: 2014/00236), CDMD (DSRB Ref: 2022/00163), and SDR (CIRB Ref: 2022/2164). No informed consent was obtained as waiver of consent was granted by DSRB and CIRB. For the NHANES study, IRB approval and informed consent were duly obtained by the NCHS-CDC (IRB: Protocol 98-12, 2005-06, 2011-17, and 2018-01). This study was conducted in accordance to the principles of the Declaration of Helsinki^29^.

### Generalized metabolic fluxes (GMF) digital twin generation and development

In this study, we utilized GMF to create personalized digital twins for each patient^6^. GMF digital twins comprise a network of dynamic variables, which represent the metabolic rates of the patient at a specific health state. We distinguished two reference health states: A) T2DM without CKD and B) T2DM with CKD. At the baseline time point, patients were either in state A or state B and using GMF, we identified and described their health state at that time point. On the other hand, during the course of our prediction study, some patients progressed from state A to state B within a defined period of time (i.e., 3 years), while others remained in state A. For each patient, the progression occurred along a single health state progression scale, which we termed a generalized extent, spanning between the two basic states: A and B. This scale depicts a continuous change in the patient’s metabolic profile. As a dynamic variable, a single individual GMF measures the rate of change of a specific metabolite concentration or a physiological reading at any given state along the progression scale. The collection of individual GMF for a particular patient at a particular time point represents that patient’s metabolic digital twin (Fig. 1, Supplementary Table 3). The GMF methodology allows us to use both complete and incomplete inputs to produce digital twins of patients and best fit models. We first evaluated the ability of GMF-based digital twins to identify CKD at baseline in two datasets (EVAS and NHANES). We then investigated the performance of GMF (complete and incomplete) in two separate datasets (SDR and CDMD) for the prediction of CKD. The completeness of inputs in each dataset is characterized in the Supplementary Materials (Supplementary Table 7). Our GMF methodology was capable of handling missing parameters without the need for imputation. We verified this by conducting the multiple imputation by chained equation (MICE) analysis with the CDMD dataset. Multiple imputation using Fully Conditional Specification (FCS) implemented by the MICE algorithm was used to impute data that were missing in this dataset. Each variable has its own imputation model and can therefore be extrapolated to fit into the other missing variables. Five imputed datasets were used to produce five sets of corresponding GMF values and this was subsequently used in the LR prediction model. The AUC obtained from these five imputed sets were then compared to the original AUC with missing parameters (Supplementary Table 8). We confirmed that using data imputation does not provide additional benefits and our GMF-based prediction model from incomplete inputs was robust enough in handling missing values.

The detailed method of producing patients GMF digital twins has been explained in our earlier technical paper^6^. For the identification of CKD, the GMF values were obtained from 10 biochemical and physiological parameters as inputs and produced 21 informative individual GMF as outputs. The outputs were combined with the patient’s age and gender (demographics) to build the LR model for the identification of CKD cases in the EVAS and NHANES datasets. Similarly, for the prediction of CKD, there were two LR models: (i) the model with complete inputs and ii) the model with incomplete inputs. For the complete model, the GMF values were obtained from 14 parameters as inputs and was tested in the SDR dataset whereas for the incomplete model, the GMF values were obtained from 11 parameters as inputs and was tested in the CDMD dataset. Both produced 21 informative individual GMF as outputs. The comprehensive list of input parameters utilized in the analysis of each dataset is shown in Table 2.

### Clinical definitions and parameter selection

Clinical measurements were taken at their point of recruitment (all datasets) and follow-up (CDMD and SDR). In the EVAS, NHANES, and CDMD datasets, patients were identified to have CKD if they fell under one of the following two categories: i) they had an Albumin to Creatinine Ratio (ACR) value of more than 3.3 mg/mmol (equivalent to 30 mg/g) ii) they had an estimated glomerular filtration rate (eGFR) value of less than 60 mL/min/1.73*m*^2^ ^12^. For the SDR dataset, we used only the second criterion: the eGFR value of less than 60 mL/min/1.73*m*^2^. This was due to the SDR dataset being primarily composed of primary care patients whereas the EVAS and CDMD datasets were primarily composed of tertiary care patients (Supplementary Fig. 1). For the EVAS and CDMD datasets, the eGFR value was calculated using the New Asian Modified CKD-EPI formula because this was the equation used by the clinician in charge of these datasets in TTSH^30^. Whereas for the NHANES and the SDR datasets, the standard Chronic Kidney Disease Epidemiology Collaboration (CKD-EPI) formula was used^31^. A complete list of abbreviations of the terms used in this study is detailed in the Supplementary Materials (Supplementary Table 9).

### Logistic regression (LR) and risk stratification analysis

We first performed the analysis on the identification of CKD disease state at baseline to determine the present health state of patients using two datasets, the EVAS and the NHANES datasets. Subsequently, we predicted the CKD disease state within three years using two separate LR models, the model with complete inputs in the SDR dataset and the model with incomplete inputs in the CDMD dataset to assess the performance of our GMF digital twins in predicting the health state of patients within 3 years. We developed the LR model using GMF derived from basic inputs (Table 2), age, and gender as predictor variables, where we plotted the receiver operating characteristic (ROC) curve and estimated the area under the curve (AUC). The final AUC was determined by calculating the median value from 2000 bootstrapping iterations for both the identification (EVAS and NHANES) and the incomplete input (CDMD) prediction models. The range of AUC values obtained from this iteration is depicted as the confidence interval (CI). In the complete parameter prediction model, the SDR dataset was randomly split into the training set (50% of the population) and two testing sets, Testing-1 (25%) and Testing-2 (25%). This randomization procedure aimed to balance key population characteristics (age, gender, hypertension status), which are significantly associated with CKD outcomes, across the training set and the two testing sets (Testing-1 and Testing-2). This was conducted to evaluate the robustness of these characteristics and to ensure the reliability of the complete parameter model’s prediction of CKD. We trained on the training set to obtain the parameters of the LR model, which was then used to predict the probability of CKD (AUC value) in the Testing-1 and Testing-2 sets.

To evaluate the efficacy of our model in stratifying patients into high-risk, moderate-risk, and low-risk groups for CKD, we compared the observed frequency of future CKD positive outcomes in each risk group to the expected frequency of CKD positive outcomes investigated in the SDR and CDMD datasets. The expected frequency of the risk groups was based on previously published risk intervals^7,8^. High-risk is classified as having > 30% CKD patients, moderate-risk as having 10–30% CKD patients and low-risk as having < 10% CKD patients.

### The correlation between observable input parameters and GMF

We examined the correlation between the input parameters and GMF together with demographic values in the CDMD dataset. Kendall’s *τ* correlation method was employed for this assessment. In cases of missing values, we substituted them with the median values from the CDMD dataset and all values were normalized before running the correlation analysis. To identify groups of correlated parameters, we utilized hierarchical clustering, using the Euclidean distance metric and the complete linkage method on the resultant correlation matrix (Fig. 2e). This was visualized using a heatmap. The actual correlation values between each input and individual GMF were presented in each cell. Stronger positive correlations were depicted by values above 0 and closer to +1 with the cells appearing more red. On the other hand, stronger negative correlations were represented by values below 0 and closer to -1 with cells appearing more blue.

### Impact of individual GMF on CKD prediction

Out of the 21 informative GMF values and the two demographic values used as predictor variables in the LR model, we identified the most significant predictors by considering the LR model coefficient weights with significant p-values. Table 5 provides a list of these significant predictor variables.

### Health states and medication effects with GMF

We then performed a metabolic profile subgroup analysis to explain the variation in the GMF metabolic profiles in different groups of patients in the CDMD dataset, based on inputs at baseline. Here, we investigated the GMF differences seen in patients who develop CKD in the future with respect to patients who do not develop CKD in the future. The grouped median values of each individual GMF within the entire GMF profile were compared in each of the subgroup analyses. The significant difference of individual GMF between the groups were evaluated using the Wilcoxon-Mann-Whitney U test with the two-sided null hypothesis that no significant difference was present (Supplementary Table 4). The GMF profiles were visualized in a standard graphical form, wherein each individual GMF within the entire GMF profile is represented as colored directed edges (Fig. 4a). The color on the map corresponds to the ratio between the median value of each individual GMF in a specific group and its median in the reference group. Elevated and reduced individual GMF that were significant in one subgroup vs the other subgroup are shown in red and blue, respectively. The individual GMF that do not vary are shown in black.

We also performed another subgroup analysis in the CDMD dataset to explain the difference in the GMF metabolic profiles of patients based on their medication status. Patient medication information was referred to identify patients taking SGLT2i. Patients had to have taken SGLT2i prior to the baseline measurement period in order to identify the effects of SGLT2i on GMF metabolic profiles. We performed a subgroup analysis to elucidate variations in GMF profiles between patients on SGLT2i and those not on SGLT2i in the future non-CKD group, using the same method explained earlier. This analysis was subsequently repeated in the future CKD group (SGLT2i vs no SGLT2i).

### GMF as a metric for CKD progression rate

We performed the time-to-first CKD event analysis in the CDMD dataset. We first expressed the parameter measurement events as a function of time using the Kaplan-Meier survival model. The GMF, along with demographic factors, was incorporated into an LR model to predict high-risk and moderate/low-risk CKD groups of patients. To compare the survival models of the patient groups, we used the log-rank statistical test. The Cox proportional hazard model was then used to predict the time of the first CKD event and the probability for a given patient to develop CKD at a given time point.

We also extended our CKD prediction to longer time windows of 5 and 10 years, utilizing the same predictive methodology in the CDMD dataset. This extension identified additional patients who would develop future CKD, thereby increasing the pool of future CKD-positive patients.

We next investigated the relationship between future CKD outcomes and clusters of patients in the CDMD dataset. To reveal associations between the groups of patients and their metabolic features, we applied k-means clustering. To determine the optimal number of clusters (k), we assessed the Between Sum of Squares (BSS) metric over a range of k values from 5 to 20. The BSS with 50% was achieved with k = 10 and this k was selected as the optimal choice for our analysis. After applying the k-means algorithm, patient clustering patterns were visualized using heatmaps. The cells in the heatmap displayed normalized values for each individual GMF or demographics (age and gender) within clusters, with elevated values appearing more red and reduced values appearing more blue. The clusters were then assigned numerical indices and ordered in ascending order of CKD outcomes rate. The cluster with the lowest fraction of future CKD-positive patients was designated as index 1. For all indices, we computed the Euclidean distance between the centroid of the i-th index and the centroid of the cluster with index 1. These distances were then plotted against the fraction of future CKD positives in each cluster, and their correlation was evaluated using Kendall’s *τ* with the two-sided null hypothesis that no correlation is present.

### Statistical analysis and computational tools

All statistical analyses were performed using R version 3.6.3. For logistic regression analysis, we utilized the glm.fit function within the core stats package in R^32^. The significant predictor variable model coefficients were obtained from the glm summary function. The ROC curves were generated using the pROC R package^33,34^. The AUC confidence interval values were also obtained from the pROC package. The Wilcoxon-Mann-Whitney U test from the core stats package in R was used to determine the significant difference of the grouped median fluxes in the different patient subgroups^33^. For the Cox proportional hazard analysis and the Kaplan–Meier survival/event estimator analysis, we used the survival R package^35^. For the correlation analysis, the cor function was used within the core stats package^33^. Subsequently, the pheatmap package was used to visualize correlation outputs^36^. The pheatmap package was also used to cluster patients with k-means and to visualize the clusters^33,36^.

### Supplementary information

Supplementary Materials

## Data Availability

The authors agree to provide the data and materials supporting the results or analyses presented in this paper upon reasonable request. Access to the SingHealth Diabetes Registry (SDR) dataset and other datasets in this paper can be granted upon reasonable request to the corresponding authors, under restrictions subject to obtaining ethics approval from institutional boards and an appropriate data-use and/or research agreement.

## Code availability

The code in this study is available from the authors upon reasonable request.
