# Supplementary material for: A digital twin model incorporating generalized metabolic fluxes to identify and predict chronic kidney disease in type 2 diabetes mellitus
Source: NPJ Digit Med. 2024 May 24;7:140. doi: 10.1038/s41746-024-01108-6 (PMC11126707; doi:10.1038/s41746-024-01108-6)
Supplement: Supplementary file 1 — Supplementary Materials [file 41746_2024_1108_MOESM1_ESM.pdf]

Supplementary Materials

Supplementary Tables

Supplementary Table 1. CKD staging categorization of GMF based future CKD risk groups in CDMD

Categories	High risk (N)	Moderate risk (N)	Low risk (N)	Total (N)
Microalbuminuria (ACR = 3.4-30 mg/mmol)	487	177	5	669
Macroalbuminuria (ACR > 30 mg/mmol)	13	3	0	16
CKD Stage G3a (eGFR = 45-59 ml/min/1.72min2)	21	8	0	29
CKD Stage G4 (eGFR = 15-29 ml/min/1.72min2)	1	0	0	1
CKD Stage 3a and microalbuminuria	4	0	0	4

Supplementary Table 2. GMF differences in subgroup analysis between future CKD vs future non-CKD

Individual GMF	Future CKD vs Future non-CKD
Erythrocytes→Haematocrit	↑
Ex.Respiration→Erythrocytes	↑
Ex.Nutrient→Glucose	↑
Albumin→ACR	↑
Liver.Protein→Albumin	↑
Ex.HbA1c→ACR	↑
Liver→Creatinine	↑
Haematocrit↔Hb	↑
Hb.ROM→HbA1c	↑
Ex.Nutrient→Protein	↑
Ex.Respiration→SBP	↑
Liver.Fat.lipids→HDL	NA
Fat.lipids↔FFA	NA
FFA→BMI	NA

Supplementary Table 3. Digital twin map metabolic pathway reference sources

Pathway	Reference Source
Respiration→Erythrocytes	Red blood cell pH, the Bohr effect, and other oxygenation-linked phenomena in blood O2 and CO2 transport.[1]
Erythrocytes→Haematocrit	Red blood cell pH, the Bohr effect, and other oxygenation-linked phenomena in blood O2 and CO2 transport.[1]
Iron→ROM	A general map of iron metabolism and tissue-specific subnetworks.[2]
Haematocrit↔Hb	Diagnostic morphology: biophysical indicators for iron-driven inflammatory diseases.[3]
Hb→Iron	Ferroptosis: Role of lipid peroxidation, iron and ferritinophagy.[4]
HDL+VLDL→LDL	The metabolic pathways of high-density lipoprotein, low-density lipoprotein, and triglycerides: a current review.[5]
Liver+Fat-lipids→HDL	High Density Lipoproteins: Metabolism, Function, and Therapeutic Potential.[6]
LDL→Cholesterol	Dyslipidaemia.[7]
VLDL→TG	The Effects of Medications Used for the Management of Diabetes and Obesity on Postprandial Lipid Metabolism.[8]
Fat-lipids↔FFA	New methodologies for studying lipid synthesis and turnover: Looking backwards to enable moving forwards.[9]
Fat-lipids→VLDL	New methodologies for studying lipid synthesis and turnover: Looking backwards to enable moving forwards.[9]
FFA→BMI	Obesity and Its Metabolic Complications: The Role of Adipokines and the Relationship between Obesity, Inflammation, Insulin Resistance, Dyslipidemia and Nonalcoholic Fatty Liver Disease.[10]
TG→BMI	Obesity and Its Metabolic Complications: The Role of Adipokines and the Relationship between Obesity, Inflammation, Insulin Resistance, Dyslipidemia and Nonalcoholic Fatty Liver Disease.[10]
Albumin→ACR	Microalbuminuria.[11]
Ex:HbA1c→ACR	Glycated albumin and glycated hemoglobin- A comparison.[12]
Ex:ROM→ACR	Diabetes-Induced Reactive Oxygen Species: Mechanism ofTheir Generation and Role in Renal Injury.[13]
Liver→Creatinine	Creatine & Creatinine Metabolism.[14]
Creatinine→eGFR	Prediction of creatinine clearance from serum creatinine.[15]
ACR→eGFR	Combining GFR and Albuminuria to Classify CKD Improves Prediction of ESRD.[16]
Liver+Protein→Albumin	Acute-phase proteins and other systemic responses to inflammation.[17]
Ex:Respiration→SBP	Effects of respiration on blood pressure and heart rate variability in humans.[18]
SBP→ACR	Relationship of Microalbuminuria with different Clinical and Biochemical Parameters in Newly Detected Diabetes Mellitus Cases.[19]
Glucose→HbA1c	Significance of HbA1c Test in Diagnosis and Prognosis of Diabetic Patients.[20]
Hb+ROM→HbA1c	Determination of glycated hemoglobin with special emphasis on biosensing method.[21]
Liver→ALT	The past and present of serum aminotransferases and the future of liver injury biomarkers.[22]
Liver→AST	The past and present of serum aminotransferases and the future of liver injury biomarkers.[22]
Ex:Nutrient→Fat-lipids	Nutrient Utilization in Humans: Metabolism Pathways.[23]
Ex:Nutrient→Protein	Nutrient Utilization in Humans: Metabolism Pathways.[23]
Ex:Nutrient→Glucose	Nutrient Utilization in Humans: Metabolism Pathways.[23]

Supplementary Table 4. Comparison between HealthVector Diabetes® against other published predictive models for CKD in T2DM populations

Predictive models in T2DM populations	Outcomes (prediction horizon)	Model input parameter list	AUC (number of patients in the validation cohort)	Sensitivity (Sn) and Specificity (Sp)
HealthVector Diabetes - Mesh Bio, Singapore	predicting the incidence of moderately to severely reduced kidney failure based on eGFR (3 years)	Age and gender plus 14 basic parameters in the GMF model (Glucose, HbA1c, Cholesterol, HDL, LDL, TG, SBP, BMI, Sr Creatinine, Sr Albumin, Haemoglobin, Haematocrit, ALT, AST)	AUC = 0.86 (n = 1,814 in testing and validation cohort)	Sn=0.80, Sp=0.74
KidneyIntelx - Renalytix,[24] USA	predicting the outcome of progressive decline in kidney function using eGFR (up to 5 years)	3 plasma biomarkers & 3 basic parameters (TNF receptors 1 and 2 (TNFR1 and TNFR2), plasma kidney injury molecule-1 (KIM-1), eGFR, uACR, and systolic BP)	AUC = 0.77 (n = 460 in validation cohort)	Sn=0.77, Sp=0.58
Shanghai Diabetes Institute,[25] China	predicting the incidence of DKD (up to 4 years)	4 basic parameters (Sex, BMI, Systolic Blood Pressure and Diabetes Duration)	AUC = 0.72 (n = 3,515 in validation cohort)	Sn=0.76, Sp=0.57
Asia University,[26] Taiwan	predicting the risk of CKD in 3 time points (1, 3, & 5 years)	11 basic parameters age, diabetes duration, insulin use, eGFR, ACR, HDL, TG, diabetes retinopathy, variation of HbA1c, variation of FPG, hypertension drug use)	AUC (average) = 0.76 (n = 1,534 in validation cohort)	Sn=0.75, Sp=0.63
National Clinical Research Center for Kidney Diseases,[27] China	predicting risk of DKD (3 years)	8 parameters (age, homocysteine, HbA1c, BMI, serum albumin, eGFR, bicarbonate, LDL)	AUC = 0.82 (n = 164 in validation cohort)	Sn=0.78, Sp=0.77
Center for Clinical Translational Innovations and Biomedical Big Data Center, Sichuan University,[28] China	predicting CKD based on eGFR and ACR (5 years)	Retinal fundus image and 6 basic parameters (age, gender, height, weight, bmi, blood pressure)	AUC = 0.72 (n = 3,376)	Not reported
QKidney validation in T2DM - University Malaya,[29] Malaysia	predicting the incidence of moderate-severe DKD (5 years)	15 basic parameters (Age, BMI, Systolic blood pressure, Smoking status, Ethnicity, Diagnosed T1DM, Diagnosed T2DM, Diagnosed RA, Diagnosed hypertension and under treatment,Diagnosed CVD, Diagnosed congestive cardiac failure, Diagnosed PVD, Diagnosed SLE, Diagnosed Renal Calculi, Positive family history of renal diseases.	AUC = 0.75 (n = 377 total patients)	Estimated Sn=0.78, Sp=0.60

Supplementary Table 5. Distribution of future CKD and future non-CKD based on ethnicity in CDMD

Ethnicity	Future CKD (N)	Future non-CKD (N)	Total (N)	χ^2 test (p-value)
Chinese	467	915	1382	
Malay	90	119	209	9.09 (0.011*)
Indian	113	252	365	

Supplementary Table 6. GMF based risk group classification based on ethnicity in CDMD

Ethnicity	High risk (N)	Moderate risk (N)	Low risk (N)	Total (N)	χ^2 test (p-value)
Chinese	624	714	44	1382	
Malay	118	87	4	209	15.34 (0.004**)
Indian	175	187	3	365	

Supplementary Table 7. Completeness of the datasets at baseline

	EVAS (N=289)	NHANES (N=1044)	CDMD (N=2112)	SDR (N=3276)
Number of patients with 0 missing values	92 (31.8%)	1022 (98%)	1228 (58%)	3627 (100%)
Number of patients with 1 missing value	132 (45.7%)	22 (2%)	884 (42%)	-
Number of patients with 2 missing values	58 (20.1%)	-	-	-
Number of patients with 3 or more missing values	7 (2.4%)	-	-	-

Supplementary Table 8. Comparison of AUC values obtained from MICE analysis against AUC derived from GMF analysis

Datasets	AUC
Original	0.7480
Imputation 1	0.7505
Imputation 2	0.7506
Imputation 3	0.7504
Imputation 4	0.7505
Imputation 5	0.7503

Supplementary Table 9. List of Abbreviations

**Abbreviations**	**Explanations**
ACR	Albumin-Creatinine Ratio
ALT	Alanine aminotransferase
AST	Aspartate aminotransferase
AUC	Area under the curve
BMI	Body mass index
CKD	Chronic Kidney Disease
eGFR	Estimated glomerular filtration rate
EMR	Electronic medical record
GMF	Generalized Metabolic Fluxes
FFA	Free fatty acids
FBG	Fasting blood glucose
Hb	Haemoglobin
HbA1c	Glycated haemoglobin
HDL	High-density lipoprotein
LDL	Low-density lipoprotein
ROC	Receiver operating characteristic
ROM	Reactive oxygen material
SBP	Systolic blood pressure
SD	Standard deviation
SN	Sensitivity
SP	Specificifty
TG	Triglyceride
VLDL	Very-low-density lipoprotein

Supplementary Figures

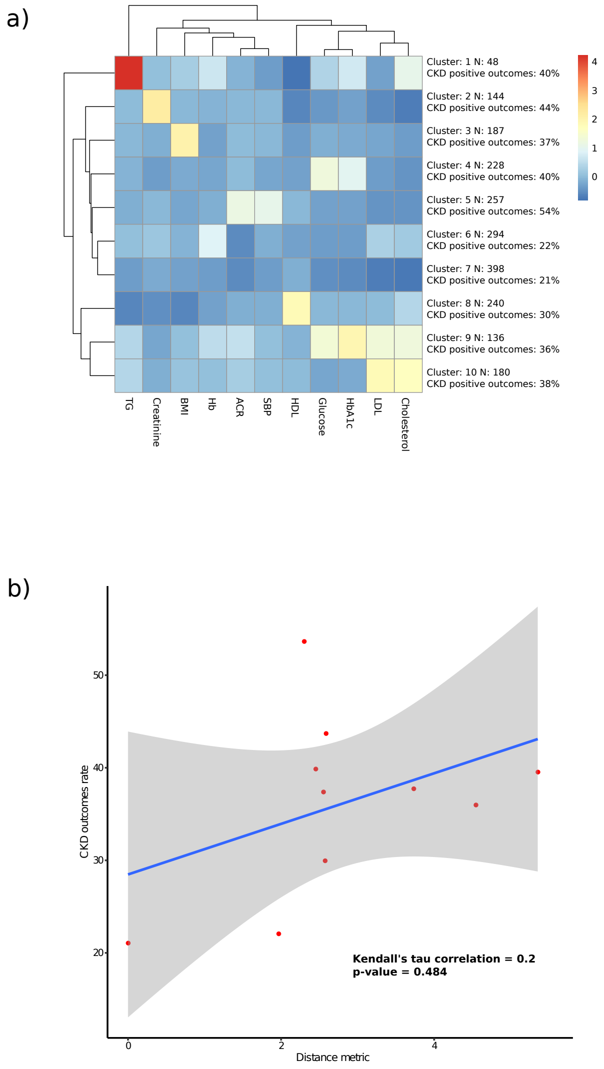

Supplementary Fig. 1. Patient clustering with inputs. **a** The clustering pattern of patients in the CDMD dataset using the inputs (clinical and physiological). **b** The relationship between the cluster distances and CKD outcomes rate. In **a**) N represents the number of patients within the specific cluster, and CKD-positive outcomes are calculated as the ratio of patients developing future CKD (within 3 years) to the total number of patients in that cluster. There is no distinct pattern observed in the cluster with the highest CKD outcomes rate. There is also no significant correlation (τ=0.2, p=0.484) between cluster distance and CKD outcomes rate.

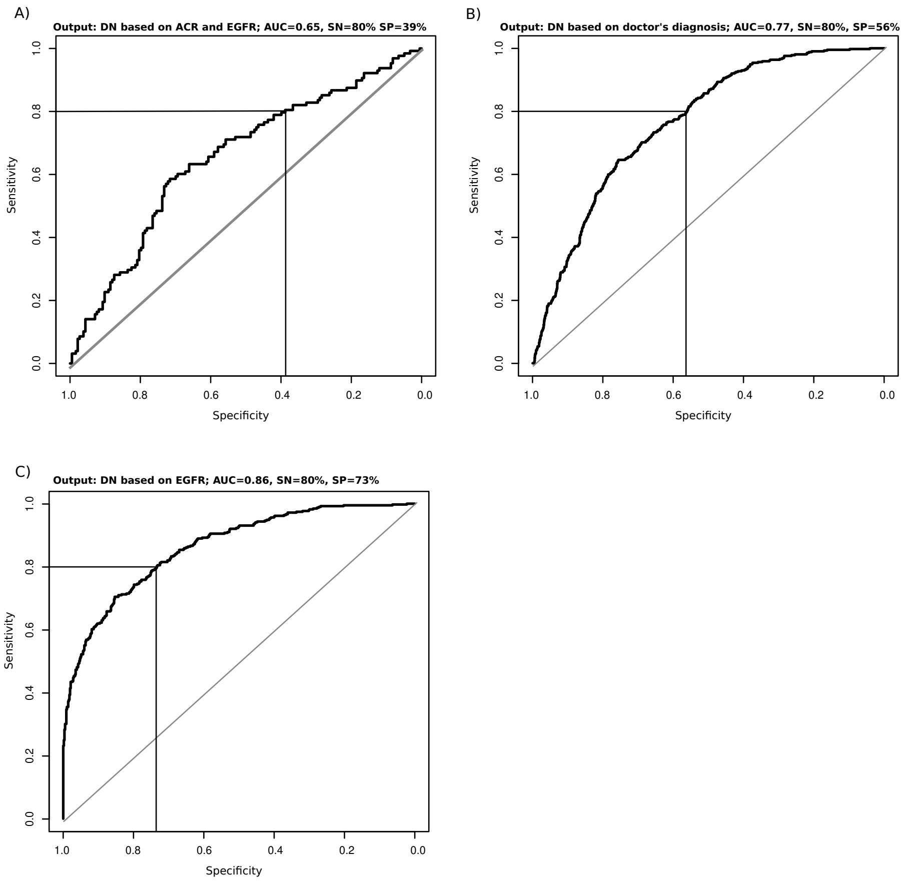

Supplementary Fig. 2. Selection of eGFR as an improved endpoint for the SDR dataset. Unlike the EVAS and the CDMD datasets, which consisted of hospital patients (the Tan Tock Seng Hosiptal), 67% of the SDR patients were from SingHealth polyclinics. In polyclinics data collection is primarily driven by the national clinical practice guidelines (CPG[30]). The Diabetes CPG recommend annual assessment of the ACR, as well as eGFR (based on serum creatinine). The CPG define the CKD status based on the eGFR values. The present study is in consistence with these quidelines. To identify the diagnostic criteria for SDR that would be most consistent with those of EVAS and CDMD, we analyzed 3 alternatives outputs for our 3-year CKD prediction model: **a**) using microalbuminuria (ACR > 3.3 mg/mMol), **b**) using the codified diagnosis record (kidney disease stage 3 or above) and **c**) eGFR < 60 mL/min/1.73. Our results indicated that using eGFR alone provided the highest performing predictive model (AUC=0.86), followed by the diagnosis records (AUC=0.77). The observed gap between the two may be explained by the explicit CPG recommendation to estimate renal function only when eGFR is below 60 mL/min/1.73, while leaving earlier stages ambiguous.

Supplementary References

[1] Jensen, F. B. Red blood cell pH, the bohr effect, and other oxygenation-linked phenomena in blood o2 and CO2 transport. *Acta Physiologica Scandinavica* **182**, 215–227 (2004).

[2] Hower, V. *et al.* A general map of iron metabolism and tissue-specific subnetworks. *Molecular BioSystems* **5**, 422 (2009).

[3] Pretorius, E. & Kell, D. B. Diagnostic morphology: biophysical indicators for iron-driven inflammatory diseases. *Integrative Biology* **6**, 486–510 (2014).

[4] Latunde-Dada, G. O. Ferroptosis: Role of lipid peroxidation, iron and ferritinophagy. *Biochimica et Biophysica Acta (BBA) - General Subjects* **1861**, 1893–1900 (2017).

[5] Kwiterovich, P. O. The metabolic pathways of high-density lipoprotein, low-density lipoprotein, and triglycerides: a current review. *The American Journal of Cardiology* **86**, 5–10 (2000).

[6] Jomard, A. & Osto, E. High density lipoproteins: Metabolism, function, and therapeutic potential. *Frontiers in Cardiovascular Medicine* **7** (2020).

[7] Durrington, P. Dyslipidaemia. *The Lancet* **362**, 717–731 (2003).

[8] Eleftheriadou, I., Grigoropoulou, P., Katsilambros, N. & Tentolouris, N. The effects of medications used for the management of diabetes and obesity on postprandial lipid metabolism. *Current Diabetes Reviews* **4**, 340–356 (2008).

[9] Previs, S. F. *et al.* New methodologies for studying lipid synthesis and turnover: Looking backwards to enable moving forwards. *Biochimica et Biophysica Acta* **1842**, 402–413 (2014).

[10] Jung, U. & Choi, M. Obesity and its metabolic complications: the role of adipokines and the relationship between obesity, inflammation, insulin resistance, dyslipidemia and nonalcoholic fatty liver disease. *International Journal of Molecular Sciences* **15**, 6184–6223 (2014).

[11] Khosla, N., Sarafidis, P. A. & Bakris, G. L. Microalbuminuria. *Clinics in Laboratory Medicine* **26**, 635–653 (2006).

[12] Mukherjee, B., Patra, S. & Das, A. K. Glycated albumin and glycated hemoglobin- a comparison. *International Journal of Biomedical Research* **4**, 381 (2013).

[13] Fakhruddin, S., Alanazi, W. & Jackson, K. E. Diabetes-induced reactive oxygen species: Mechanism of their generation and role in renal injury. *Journal of Diabetes Research* **2017**, 1–30 (2017).

[14] Wyss, M. & Kaddurah-Daouk, R. Creatine and creatinine metabolism. *Physiological Reviews* **80**, 1107–1213 (2000).

[15] Cockcroft, D. W. & Gault, H. Prediction of creatinine clearance from serum creatinine. *Nephron* **16**, 31–41 (1976).

[16] Hallan, S. I. *et al.* Combining GFR and albuminuria to classify CKD improves prediction of ESRD. *Journal of the American Society of Nephrology* **20**, 1069–1077 (2009).

[17] Gabay, C. & Kushner, I. Acute-phase proteins and other systemic responses to inflammation. *New England Journal of Medicine* **340**, 448–454 (1999).

[18] Elghozi, J.-L., Laude, D. & Girard, A. Effects of Respiration on Blood Pressure and Heart Rate Variability in Humans. *Clinical and Experimental Pharmacology and Physiology* **18**, 735–742 (1991).

[19] Prasad, I. *et al.* Relationship of microalbuminuria with different clinical and biochemical parameters in newly detected diabetes mellitus cases. *Ibrahim Medical College Journal* **4**, 21 (2010).

[20] Sherwani, S. I., Khan, H. A., Ekhzaimy, A., Masood, A. & Sakharkar, M. K. Significance of HbA1c test in diagnosis and prognosis of diabetic patients. *Biomarker Insights* **11**, BMI.S38440 (2016).

[21] Pundir, C. S. & Chawla, S. Determination of glycated hemoglobin with special emphasis on biosensing methods. *Analytical Biochemistry* **444**, 47–56 (2014).

[22] McGill, M. R. The past and present of serum aminotransferases and the future of liver injury biomarkers. *Experimental and Clinical Sciences* (2016).

[23] Da Poian, A. T., El-Bacha, T. & Luz, M. R. Nutrient utilization in humans: Metabolism pathways. *Nature Education* **5**, 11 (2010).

[24] Chan, L. *et al.* Derivation and validation of a machine learning risk score using biomarker and electronic patient data to predict progression of diabetic kidney disease. *Diabetologia* **64**, 1504–1515 (2021).

[25] Wu, M. *et al.* A non-laboratory-based risk score for predicting diabetic kidney disease in chinese patients with type 2 diabetes. *Oncotarget* **8**, 102550–102558 (2017).

[26] Lin, C.-C. *et al.* Development and validation of a risk prediction model for chronic kidney disease among individuals with type 2 diabetes. *Scientific Reports* **12** (2022).

[27] Dong, W. *et al.* Prediction models and nomograms for 10-year risk of end-stage renal disease in chinese type 2 diabetes mellitus patients in primary care. *Diabetes, Obesity and Metabolism* **23**, 897–909 (2021).

[28] Zhang, K. *et al.* Deep-learning models for the detection and incidence prediction of chronic kidney disease and type 2 diabetes from retinal fundus images. *Nature Biomedical Engineering* **5**, 533–545 (2021).

[29] Sheng Qian, Y. & Moy, F.-M. Predicting the risk of chronic kidney disease among type 2 diabetes mellitus patients in a primary care setting: An evaluation of the qkidney model. *Malaysian Journal of Medicine and Health Sciences* **15**, 67–73 (2019).

[30] Goh, S. *et al.* Ministry of health clinical practice guidelines: Diabetes mellitus. *Singapore Medical Journal* **55** (2014).
